# Sharp turning maneuvers with avian-inspired wing and tail morphing

**DOI:** 10.1038/s44172-022-00035-2

**Published:** 2022-11-24

**Authors:** Enrico Ajanic, Mir Feroskhan, Valentin Wüest, Dario Floreano

**Affiliations:** 1 School of Engineering, Ecole Polytechnique Fédérale de Lausanne, Lausanne, Switzerland; 2 School of Mechanical and Aerospace Engineering, Nanyang Technological University, Singapore, Singapore

**Keywords:** Mechanical engineering, Biomimetics

## Abstract

Flight in dense environments, such as forests and cities requires drones to perform sharp turns. Although fixed-wing drones are aerodynamically and energetically more efficient than multicopters, they require a comparatively larger area to turn and thus are not suitable for fast flight in confined spaces. To improve the turning performance of winged drones, here we propose to adopt an avian-inspired strategy of wing folding and pitching combined with a folding and deflecting tail. We experiment in wind tunnel and flight tests how such morphing capabilities increase the roll rate and decrease the turn radius - two measures used for assessing turn performance. Our results indicate that asymmetric wing pitching outperforms asymmetric folding when rolling during cruise flight. Furthermore, the ability to symmetrically morph the wing and tail increases the lift force, which notably decreases the turn radius. These findings pave the way for a new generation of drones that use bird-like morphing strategies combined with a conventional propeller-driven thrust to enable aerodynamic efficient and agile flight in open and confined spaces.

## Abstract (web summary)

Fixed wing drone flight in dense (urban or forest) environments is challenging due to a need for a large area to turn. Inspired by the avian wing morphing, Enrico Ajanic and colleagues proposed and tested a drone with wings capable of folding and pitching, and a tail capable of folding and deflecting as a strategy to increase the roll moment, lift force, and reduce the turn radius. This finding enables agile drone flight within limited space.

## Introduction

Multi-copters are often used to fly in dense environments, such as cities, because they display higher agility, albeit at the cost of substantially lower flight endurance, compared to fixed-wing drones of similar mass. Fixed-wing drones, instead, are used in missions that require high endurance and range, such as survey of agriculture or mapping. However, they need a comparatively larger space to turn and thus fly only in open spaces^1^. Here, we show that avian-inspired folding and pitching wings substantially improve the turning capability of fixed-wing drones with propeller-driven thrust.

**Fig. 1:**
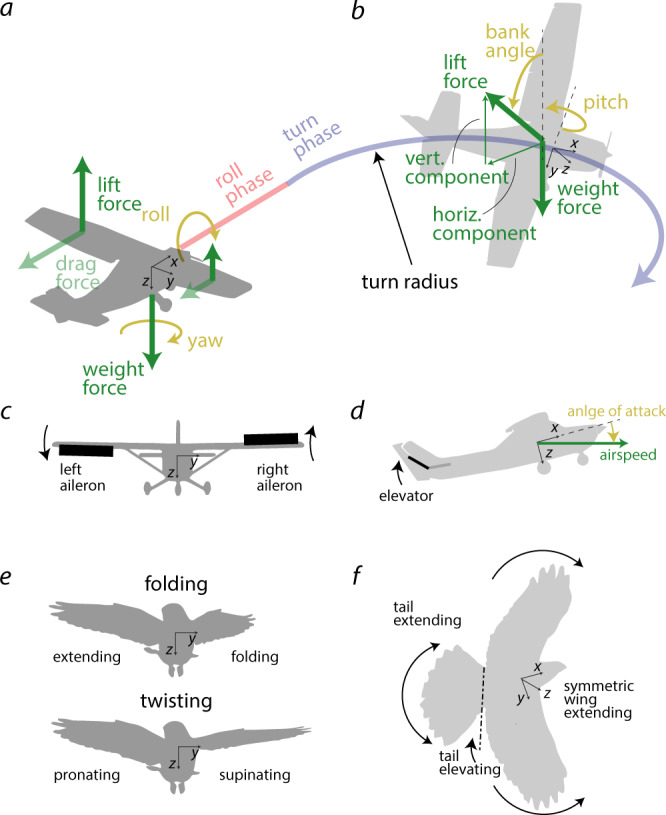
Comparing drones’ and birds’ strategies to perform a turn maneuver. **a** During the roll phase, the aircraft produces a roll motion by generating a lift asymmetry between the two wing sides until they reach their desired bank angle. **b** During the bank phase, banking results in a vertical lift component, which counterbalances the weight force and an inward facing, horizontal lift component which leads to turning. **c** To roll, drones commonly use ailerons, flaps that are deflected in opposition to each other. **d** To increase lift when turning, drones deflect their elevator upward, which increases the angle of attack, and hence the lift force. **e** Birds apply two main strategies for rolling. They use folding by extending one wing side and folding the other, or use twisting by pronating one wing side and supinating the other. **f** When turning, birds increase the lift force by elevating their tail and extending both wing and tail.

A turn maneuver can be separated into two phases: a roll phase (Fig. 1a) and a bank phase (Fig. 1b). During the roll phase, drones increase their bank angle by applying a roll moment^2^. The roll performance is best when the time to reach a desired bank angle is minimized. Once the desired bank angle is reached, the bank phase begins. When banking, the lift vector is composed of a vertical and a horizontal component. The vertical lift component must be equal to the drone’s weight force to maintain altitude, while the horizontal lift component creates the centripetal force that causes a turn motion. Increasing the lift vector when banking requires an increase in bank angle (given a constant weight force and vertical lift component), which leads to a larger centripetal force and thus a smaller turn radius (Fig. 1a). The bank phase performance is best when the turn radius is minimized, which requires lift to be maximized. Thus, the overall turn performance is maximized when both the roll time is minimized and the lift vector is maximized.

Conventional drones initiate the roll phase with ailerons, which are flaps on the trailing edge of the wings that are deflected in opposition to one another (Fig. 1c). Lowering the aileron increases lift, whereas raising the aileron decreases lift. The force asymmetry between the two wing sides causes a roll moment which translates into a roll motion. Ailerons are widely applied to today’s winged drones because of their simple mechanical design and aerodynamic efficiency^3^. During the bank phase, winged drones usually maintain a constant bank angle until the desired heading is reached. To improve the turn performance when banking, drones deflect the tail elevator upward (Fig. 1d), which increases the wing’s angle of attack and thus the overall lift to reduce the turning radius.

Gliding birds can perform sharp course variations that outperform current winged drones^4,5^ by modifying the shape and inclination of their wing and tail^6–9^. For rolling, birds can resort to two strategies: asymmetric wing folding and wing twisting. Wing folding is predominantly achieved by flexing the wrists (Fig. 1e, top), and results in a reduction of the wing area^10^. The lift force produced by each wing side is linearly dependent on the wing area^2^. Folding only one side of the wing produces an asymmetric lift distribution along the wing span that results in a roll motion. Furthermore, reducing the wing span lowers the flight inertia and enables the bird to reach the desired bank angle faster^11^. The second strategy consists in actively twisting the wings (Fig. 1e, bottom), that is changing the incidence angle of each wing side, which is achieved by pronating or supinating the wrists^12^. Increasing the wing incidence angle (pronating) increases lift, whereas decreasing the wing incidence angle (supinating) decreases lift. Pronating one wrist and supinating the other generates an asymmetric lift distribution along the wing span, resulting in a roll motion. Birds may use both strategies, wing folding and wing twisting, independently or in combination to achieve rapid roll^13^, however, the aerodynamic impact of wing twisting and folding when turning in gliding birds is still not fully understood.

Once the bank angle is reached, birds can benefit from symmetric wing and tail morphing in order to reduce the turning radius^14^ (Fig. 1f). Extending wing and tail leads to an increase in aerial surface and thus in lift; furthermore, sweeping the wing forward and enlarging the tail results in a higher angle of attack, which further increases the overall lift^13,15^. The lift increase from wing and tail morphing allows birds to fly at higher bank angles, thus reducing their turn radius.

Avian-inspired, asymmetric wing folding^16–19^ or asymmetric wing twisting^20–22^ have recently been applied to winged drones and tested in flight. Wing twisting can be an effective way to bank, and a means to control roll in the post-stall regime (complex flight condition often accompanied by a loss of control beyond the critical angle of attack) when applied only in the outer regions of the wing^20^. It has been shown that asymmetric wing folding can be used to control roll^16,18^ or to maintain a steady bank angle^17^. In our previous study with an avian-inspired drone capable of morphing both wing and tail^18^, we noticed that rolling by asymmetric folding of the wings must be preceded by a slight increase of angle of attack of the drone in order to produce a turning behavior. This insight led us to suspect that the drone could have profited from the ability of slightly twisting the wings as birds can do. However, to date, the influence of wing folding, wing twisting, and tail folding on turning performance drones and birds remains largely unknown.

Here, we systematically assess the relative impact of wing folding and twisting on rolling performance, as wells as the impact of symmetric wing and tail extension to increase lift and reduce turning angle during the banking phase, for rapid turning maneuvers. Our study relies on a bird-like morphing drone, which is an evolution of our previously feathered drone^18^ by including the ability to individually pitch the two sides of the main wings similar to bird wings. To estimate how asymmetric folding and pitching compare during the rolling phase, we conducted wind tunnel tests to measure the roll moment and also performed roll flight tests. We show that asymmetric wing pitching leads to notably larger roll moments than asymmetric wing folding in cruise flight, leading to high roll rates that enable the drone to reach the desired bank angle faster. To estimate how symmetric wing and tail morphing can reduce the turn radius during a banking turn, we developed a steady turn model (Eq. (16)) of the drone based on wind tunnel measurements and compared the model to the flight test data. The results indicate that symmetrically extending the wing and tail leads to a reduction in turning radius by more than two times.

## Results

### Avian-inspired drone

**Fig. 2:**
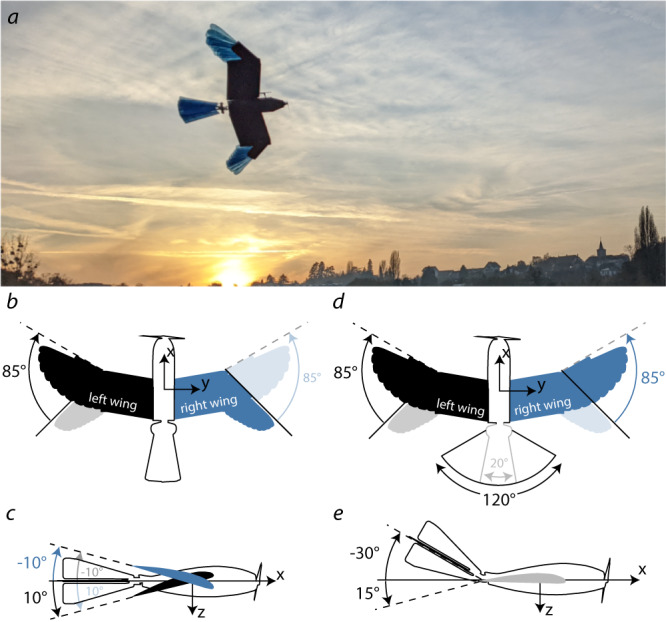
The LisEagle drone and its morphing capabilities. **a** LisEagle drone in cruise flight with a tucked wing and tail. To produce a roll moment, the LisEagle drone can either **b** asymmetrically fold its wings, or **c** pitch its wings asymmetrically (clockwise rolling: solid colored; counter-clockwise: light colored). To increase the overall lift force during the bank phase, the LisEagle drone can **d** increase its wing and tail area from tucked (light colored) to extended (solid colored) and **e** deflect the tail upward.

We developed a feathered drone, called LisEagle (Fig. 2a), which builds upon a previous prototype with a feathered morphing wing and tail^18^. We extended this design by adding a wing pitching mechanism to imitate the wing twisting seen on birds^8^. The drone has a maximum wing span of 1.52 m and a ready-to-fly mass of 711 g (see “Methods” for detailed design description).

Like birds, our LisEagle drone can independently change the sweeping angle of the outer wing sections made of artificial feathers (Fig. 2b and d; see Supplementary Method 1 and Supplementary Fig. S1A for response time). Changing the sweep angle allows the drone to change the wing area and shape in flight. Also, the drone can independently change the incidence angle of each wing side (equal variation of incidence angle along the entire length of the wing), which we call wing pitching (Fig. 2c; see Supplementary Fig. S1B for response time). Furthermore, the LisEagle possesses a morphing tail made from artificial feathers that can change its area by fanning in and out (Fig. 2d) and can deflect in the x-z-plane analogous to the elevator of a conventional aircraft (Fig. 2e).

To roll, the LisEagle either folds or pitches its wings asymmetrically. When folding, one wing side is extended by a maximum of 85° and the other remains tucked (Fig. 2b) resulting in a larger lifting surface on one wing side. Since lift is proportional to the lifting surface^2^, the extended wing side produces more lift than the tucked wing side, which causes a roll motion. When pitching (±10°), the incidence angle of one wing is increased while the other is decreased (Fig. 2c). The resulting difference in angle of attack between both wing sides (max. 20°) causes a roll motion.

To increase the lift during the turn, the LisEagle can apply two avian-inspired morphing strategies: (i) tail elevation, and (ii) wing and tail extension. The tail can be continuously deflected between a range of −30° (upward) and 15° (downward). Tail elevation produces a positive pitch moment (Fig. 1b), which increases the drone’s angle of attack and thus the lift force. Extending both wing sides by 85° and the tail by 120° (Fig. 2d) leads to an increase in total aerial surface (41%) and a large positive pitch moment. The combination of tail deflection and wing and tail extension enables the drone to fly at high angles of attack while generating large lift forces.

### Roll phase

**Fig. 3:**
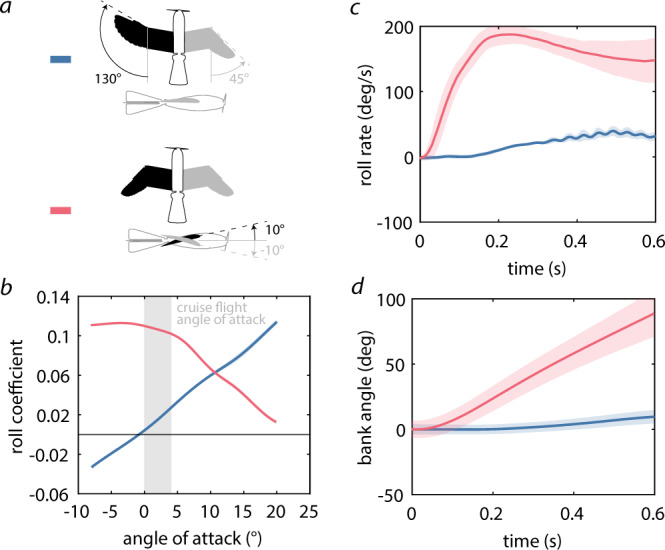
Roll performance for two different wing and tail configurations. The gray shaded area indicates the expected cruise angle of attack range. The red and blue shaded areas indicate the standard deviation from four trials (*n* = 4). **a** We tested two configurations: asymmetric wing folding and asymmetric wing pitching. **b** Angle of attack dependent roll coefficient (cubic spline, see Supplementary Fig. S3 for data points and error bars) for asymmetric wing folding (blue) and pitching (red) obtained with wind tunnel tests. **c** Time variant roll rates for asymmetric wing folding (blue) and pitching (red) measured during outdoor flight tests. **d** Time variant bank angles for asymmetric wing folding (blue) and pitching (red) measured during outdoor flight tests.

Initiating a rapid turn maneuver requires a fast transition from cruise flight (tucked wing and tail) at zero bank angle to the desired bank angle for the banking turn (Fig. 1a). The only external variable affecting the roll motion is the aerodynamic roll moment induced by the modification of the aerial surfaces. To decrease the time to reach the desired bank angle, and thus improve the roll performance, it is necessary to produce a large roll moment. To estimate the roll performance of asymmetric wing folding and pitching, we compared the roll moment coefficients (Eq. (1)) of two drone configurations: (i) we extended the left wing while keeping the the right wing tucked, without applying wing pitching (Fig. 3a, upper), and (ii) we pitched the left wing downward by 10° and the right wing upward by 10°, while keeping both wings tucked (Fig. 3a, lower). We obtained the roll coefficients through wind tunnel measurements, which we performed at angles of attack between −8° and 20° in 4° steps with cubic interpolations at an airspeed of 10 m/s.

Experimental results revealed that asymmetric wing folding generates a low roll coefficients (0.016) during cruising flight (between 2° and 8° angle of attack), although the roll coefficient steadily rises with increasing angles of attack (Fig. 3b, blue line). In comparison, pitching the two tucked wings generates a substantially larger roll moment coefficient (0.079) in cruising flight, although it rapidly decreases with increasing angles of attack (Fig. 3b, red line).

#### Rolling in flight experiments

To validate the roll performance results obtained in the wind tunnel, we performed a set of roll maneuvers with the drone flying outside. Each maneuver was initiated at cruise flight (tucked wing and tucked tail) at 60% throttle (airspeed: ~12 m/s) by switching into the configurations shown in Fig. 3a, while maintaining the same flight direction through manual elevator and rudder inputs, thus approximately maintaining the cruise angle of attack regime. We performed four roll maneuvers for each configuration.

The flight results (Fig. 3c and d) validate the wind tunnel measurements (Fig. 3b). Asymmetric sweeping of the wing during flight leads to a peak roll rate of 37°/s (Fig. 3c, blue line) and a change in bank angle of 18° after 0.6 s (Fig. 3d, blue line). In comparison, pitching the wing leads to a peak roll rate of 185°/s (Fig. 3c, red line), which translates into a change in bank angle of 92° after 0.6 s (Fig. 3d, red line). Overall, the flight results indicate that wing pitching can increase the bank angle 5.1 times more than wing folding within 0.6 s.

### Bank phase

**Fig. 4:**
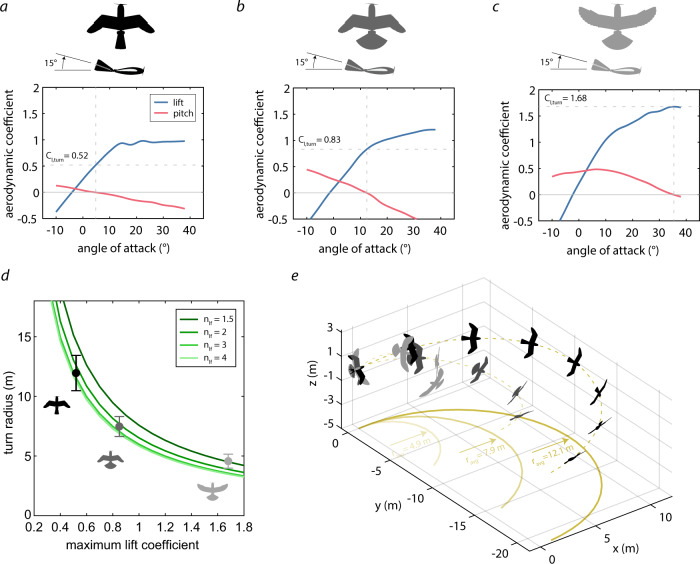
Banking performance for three different wing and tail configurations. **a**–**c** Cubic interpolation of the lift and pitch coefficient (see Supplementary Fig. S4 for the data points and error bars) with **a** tucked wing and tail, **b** tucked wing, extended tail, **c** extended wing and tail, while deflecting the tail −15° upward. **d** Turn radius as a function of maximum lift coefficient obtained with the steady-bank turn model (Eq. (16)). *n*_*lf*_ is the load factor (lift divided by aircraft weight). We have also added the mean turn radius (dots) and standard deviation (error bars) for tucked wing and tail (black), tucked wing and extended tail (gray), and extended wing and tail (light gray) from our outdoor flight tests for comparison (*n* = 5). **e** Turn trajectories and drone attitude (see Supplementary Fig. S5 for mean bank angles and air speed) of the three different wing and tail configurations during outdoor flight tests. The yellow dashed lines represents one representative trial run, the solid yellow lines are their projections on the *xy*-plane, and the average radii are the mean radius from 5 flown turns (*n* = 5). For sake of clarity, the drone is depicted 2x larger than its real size.

Turning with a small radius requires large lift forces. To identify how wing and tail morphing can increase lift when banking, we performed wind tunnel tests and measured the lift and pitch moment for different angles of attack in three configurations: tucked wing and tail (Fig. 4a), tucked wing and extended tail (Fig. 4b), and extended wing and extended tail (Fig. 4c), while deflecting the tail upward by 15°. During a steady banking turn, the drone is in equilibrium and the sum of all forces and moments are zero. Thus, it follows that the pitch moment coefficient (Eq. (5)) must be zero (Fig. 4a to c, crossing of blue line and horizontal Gray line)^2^. Drawing a vertical line through the zero-pitch location we could determine the bank angle of attack and the the corresponding lift coefficient (Fig. 4a to c, crossing of red line and vertical dashed line; Eq. (4)).

When tucking wing and tail while deflecting the tail upward by 15°, our measurements indicate a pitch equilibrium at an angle of attack of 4.5° leading to a lift coefficient of 0.52 (Fig. 4a). When maintaining a tucked wing while extending the tail and deflecting the tail upward by 15°, the equilibrium angle of attack is raised to 12.5°, thus increasing the lift coefficient to 0.83 (Fig. 4b). When extending wing and tail and deflecting the tail upward by 15°, the pitch equilibrium is at 38° angle of attack, which notably increases the lift coefficient to 1.68 (Fig. 4c). We can expect a notable reduction in turn radius when extending wing and tail resulting from the lift increase (factor of 3.2), given the connection mentioned above between lift increase and turn radius reduction.

#### Steady banking turn model

To analyze how the aforementioned changes in lift coefficients affect the turn radius (and to potentially compare its turn performance with other drones, see Supplementary Note S2 and Fig. 6), we developed a steady banking turn model (Eq. (16)) based on^2^, which relates the lift coefficient to the turn radius (Fig. 4d; see “Methods”).

The model predicts that for low lift coefficients (for example the 0.52 measured for the tucked wing and tail configuration, Fig. 4a) the drone must perform turns with a radius 12 m in order to compensate for the downward force of its own weight (Fig. 4d). Increasing the lift coefficient decreases the turn radius. For example, at a lift coefficient of 1.68 (Fig. 4c), the minimum turn radius is around 4.8 m. Another factor affecting the turn radius is the load factor *n*_*lf*_, which is the maximum expected lift force divided by the drone weight^2^. The turn radius decreases with increasing load factors. For example, at a lift coefficient of 1, increasing the load factor from 1.5 to 4 decreases the turn radius from 7.6 to 5.8 m, respectively. We designed the LisEagle’s wing and tail to withstand a load factor of 4.

#### Bank turning in flight experiments

To compare our steady turn model to the drone’s real-world flight behavior, we conducted outdoor banking turn flight tests with the LisEagle drone (Supplementary Vid. S1). The banking turn maneuver was initiated from cruise flight at 60% throttle (resulting in a speed of ~12 m/s, Supplementary Fig. S5D) by pitching the tucked wings asymmetrically. Once the drone was banked (see Supplementary Fig. S5C for mean bank angles), we initiated the turn by switching into one of the three configurations depicted in Fig. 4a to c, while maintaining a throttle setting of 60%. For each configuration, we considered five turn maneuvers (see “Methods”), from which we calculated the mean turn radius (Fig. 4d, e) and the corresponding standard deviation (Fig. 4d).

The mean turn radius for the tucked wing and tail configuration (12.1 m) lies within the predicted turn radius (11.2–14.6 m) (Fig. 4d, e, black). The standard deviation was highest for this experiment (1.2 m), which can be explained by the longer flight path, and hence the greater possible deviation from a steady level turn. The mean bank angle measured in these turn maneuver was 49°, which was the lowest of the three configurations (Supplementary Fig. S5C). The mean radius for the tucked wing and extended tail flight tests (7.9 m) correlated well with the radius predicted by our model (6.9–8.9 m) (Fig. 4d, e, gray). The standard deviation of the experiments was 1.0 m. The mean bank angle measured in these turn maneuvers was 53° (Supplementary Fig. S5C). The mean turn radius for the extended wing and tail configuration (4.9 m) was slightly higher than the modeled turn radius (3.6–5.0 m) (Fig. 4d and e, light gray), and the standard deviation was the smallest (0.5 m). As expected, the mean bank angle obtained from these turn maneuvers was the largest at 78° (Supplementary Fig. S5C). Unfortunately, we could not calculate the turn load factors for the three configurations, which would require the knowledge of the time resolved angle of attack and angle of side slip angle^2^. However, considering the body’s g-force (mean of the five trial runs) as a conservative estimate of the load factor (Supplementary Fig. S5B), we see that the peak g-force always remains well below the aforementioned design load factor of 4 for all configurations. The peak g-force experienced by the drone when in the tucked wing and tail configurations is 1.46, while when extending wing and tail the peak g-force reaches 3.3.

## Discussion

We have developed an avian-inspired morphing drone with a wing capable of folding and pitching, and a tail capable of folding and deflecting, in order to test to identify bird-like morphing maneuvers that lead to higher turn performance. The drone improves from our previous morphing drone^18^ by including a wing pitching mechanism. Unlike the bird’s pitching wing, which increases the wing incidence towards the wing tip by supinating or pronating its wrist^23^, the LisEagle rotates the entire wing resulting in the same incidence change along the entire wing span (see “Methods”). While this design choice required a simple mechanism (see “Methods”) because the entire wing could be designed as a stiff element, the uniform rotation of the wing meant that the angle of attack increases along the wing span when rolling, which may lead to stall conditions at the wing tips^12^ and thus a lower roll performance compared to birds.

Our study highlights the limitations of asymmetric wing folding to control roll during cruise flight. In previous studies, asymmetric wing folding was successfully used to roll^16,17^, but the angles of attack were not reported in those studies. Our flight tests show that asymmetric wing folding is not very effective to roll the drone in cruise flight (angles of attack in the range from 2° to 4°). In comparison, asymmetric wing pitching resulted in 426% higher roll rate (Fig. 3c), i.e. reaching a much higher bank angle within a given amount of time. Our drone could reach a bank angle of only 10° in 0.4 s° with asymmetric folding and of 60° with asymmetric wing pitching within the same amount of time (Fig. 3d). Here, we would like to highlight that asymmetric wing folding could generate a higher roll performance if the area difference between the two wing sides is larger than that tested in these experiments (31% difference), if the drone flies at higher angles of attack during cruise flight (e.g., at lower flight speeds), or if the moment of inertia in roll is reduced (e.g., reducing the mass of the wings).

We noticed that the roll moment for wing pitching decreases with increasing angles of attack (Fig. 3b, red line), which is a well-known trend also in conventional aircraft with ailerons. This is because at higher angles of attack, increasing the wing incidence no longer leads to an increasing lift force, and it may even lead to a decreasing lift force, resulting in a decrease (Fig. 2b) or even reversal of the roll moment^3^. However, our results show that wing folding produces an increasing roll moment with increasing angles of attack (Fig. 3b, blue line). This can be explained by the difference in wing area between the two wing sides, which will always produce a lift difference at increasing angles of attack. This finding suggests that asymmetric wing folding leads to higher roll rates at high angles of attack than asymmetric wing pitching. A systematic comparison of the roll performance of folding and pitching at increased angles of attack including flight tests will be a future research avenue. These tests will require precise high angle of attack feedback control on the LisEagle during flight, which was not available in this project.

Producing a roll moment often leads to adverse yaw^24^, which is an opposing moment about the aircraft’s z-axis (Supplementary Fig. S2A, left, and Supplementary Note S1) and causes slipping whereby the nose of the aircraft no longer points in the direction of travel. The slipping results in a reduction of centripetal force and increased drag that decreases turn performance (Supplementary Fig. S2A, right). Our wind tunnel measurements and flight tests indicate a similar adverse yaw moment and motion, respectively, with asymmetric wing folding and pitching in cruise flight (Supplementary Fig. S2C to E). However, while our wind tunnel measurements indicate an increase in adverse yaw moment with asymmetric wing pitching at higher angles of attack, the adverse yaw moment remains nearly constant with asymmetric wing folding at increasing angles of attack (Supplementary Fig. S2C). This finding leads to the hypothesis that asymmetric wing folding might be more favorable in rolling at higher angles of attack to minimize adverse yaw effects.

It is not yet fully understood why birds use asymmetric folding and twisting to initiate rolling in gliding flight^13,25^. Our roll phase results with the LisEagle drone may explain when birds apply wing folding or twisting. Analogous to our aforementioned results, we hypothesize that birds may predominantly rely on asymmetric wing twisting to roll in cruise flight (low angles of attack around 0 to 10°), achieving a high roll moment and a low adverse yaw moment (Fig. 3 and Supplementary Fig. S2). When flying at higher angles of attack (beyond 10°), birds may revert to asymmetric wing folding, which produces a greater roll moment and a lower adverse yaw moment, as compared to asymmetric twisting. However, although our drone is similar in size, weight, and morphing capabilities to real birds, in vivo experiments will be needed to confirm these hypotheses.

The bank phase flight experiments indicated that symmetric wing and tail morphing can reduce the turn radius by 56.5% from 12.1 m when the wings and tail are tucked to 4.9 m when the wings and tail are extended (Fig. 4e). The turn radius reduction resulted from the increased lift force generated by the extended wing and tail (lift coefficient: 1.68) compared to the tucked wing and tail (lift coefficient: 0.52). Although the flight experiments were not perfectly steady (Supplementary Fig. S5), the measured turning radii are in line with those predicted by our banking turn model. The model assumes an ideal, steady-level turn with a constant bank angle throughout the maneuver and zero side-slip. In contrast, during outdoor flight airspeed, bank angle, side-slip angle, and altitude varied (especially for the extended wing, extended tail configurations) due to pilot reaction times and wind causing changing bank angles, and increased drag when extending the wing (leading to a speed reduction) throughout the measured range (Supplementary Fig. S5), which leads to discrepancies between the model and the flight tests (Fig. 4d).

Overall, the roll and the bank phase results suggest that the synergistic wing and tail morphing substantially improve the drone’s capability to perform sharp course variations and reduce the required space. For example, given a bank angle of 50° and a cruise speed of 12 m/s, a drone pitching the wings would cover 5 m from the roll initiation until the bank angle is reached, followed by a turn with extended wing and tail with a radius of 4.9 m. In total, the drone would require 9.9 m to perform a complete 180° turn maneuver. In comparison, a drone folding the wings to roll and keeping wing and tail tucked in during the turn, would require about 22.9 m to achieve a bank angle of 50° (extrapolated from our roll flight test data, Fig. 3d) followed by a turn with a turn radius of 12.1 m. Thus, the entire turn maneuver would require 35 m.

The higher roll performance of asymmetric pitching compared to folding when cruising, and the increased turn performance of symmetric wing and tail morphing presented here show the potential of avian-inspired morphing in sharp turning maneuvers. These results can path a way to a new type of winged drone capable of effortlessly negotiating cluttered environments as well as fly long distances, while using conventional propellers for thrust generation.

## Methods

### Morphing drone

**Fig. 5:**
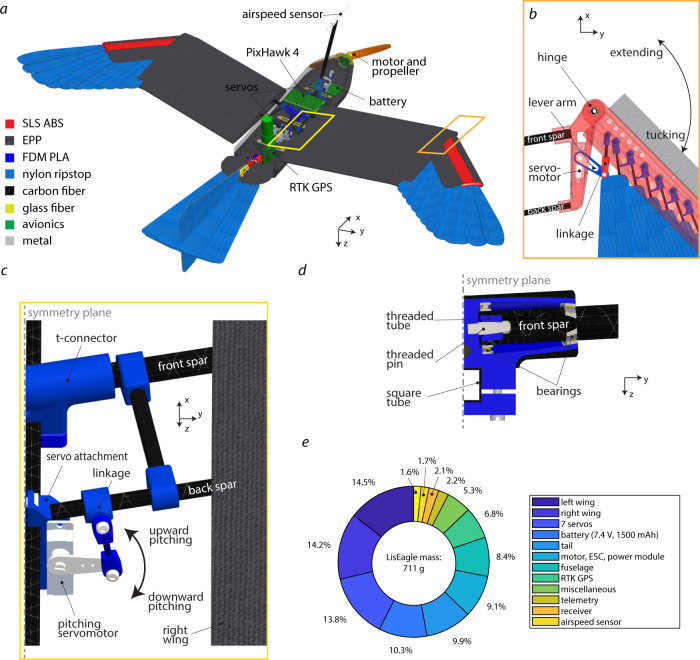
Mechanical design of the LisEagle drone. **a** Overview of the main materials and avionics used for the LisEagle drone. **b** Detailed illustration of the wrist design and actuation mechanism. For sake of clarity, we removed the EPP wing. **c** Detailed illustration of the pitching mechanism. For sake of clarity, we removed the EPP fuselage. **d** Cut of the t-connector permitting the pitching of the wing. **e** Proportion of weight for the LisEagle’s main components.

The LisEagle drone is made of four main material groups: 3d printed thermoplastics such as acrylonitrile butadiene styrene (ABS) and polyactide (PLA), flexible foam such as expanded polypropylene (EPP), fiber reinforced plastics such as carbon and glass fibers, and a thin and woven nylon membrane (Fig. 5a). As avionics we used a T-Motor AT-2306 KV2300 brushless outrunner motor in combination with a 8 × 4 inch GWS propeller (measured nominal thrust at 7.4 V was 5.2 N), a T-motor F30A ESC and a 2S lithium polymer battery with a capacity of 1500 mAh. All morphing surfaces are actuated by a KST X08plus servomotor. To log the drone’s attitude, position, and airspeed, and control outputs, we installed a PixHawk 4 autopilot (logging rate: 250 Hz for raw acceleration and angular rates), a Drotek RTK GPS (logging rate: 10 Hz, accuracy: <0.02 m), and a Sensirion SDP3x airspeed sensor (logging rate: 100 Hz, accuracy: 0.2 m/s above 5 m/s). We used the Turnigy X9R remote control to teleoperate the drone during flight. The weight percentages of the different drone parts are shown in Fig. 5e.

To retain the wing folding from the previous design in^18^ together with the wing pitching, we fixed the two wing folding servomotors (KST X08plus) within the hinge part on the wing (Fig. 5b). The servo’s increased lever arm is directly connected to the wrist through a linkage made from a carbon reinforced plastics and PLA. The right wing is extended through a counter clockwise servo rotation and rucked through a clockwise servo rotation (FIG). The right wing is extended through a counter-clockwise rotation of the servomotor and tucked through a clockwise rotation of the servomotor. Conversely, the left wing is extended through a clockwise rotation of the servomotor, and tucked through a counter-clockwise rotation of the servomotor.

The central piece of the pitching mechanism is carbon-fiber reinforced t-connector made from PLA, which connects the wings to the fuselage and absorbs most of the wing forces and moments (Fig. 5c). Two ball bearings (dimensions: 12–8–2 mm) are positioned on each side of the t-connector’s arms to hold the front spar of the wing (Fig. 5d). The spar is fixed by a threaded pin through a threaded hole installed at the end of the wing spar. While this locks the fuselage with the wing axially, the wing can still rotate around the front spar, thus allowing variable incidence angle. The wing pitching mechanism is actuated by two servomotors (KST X08plus)—one servo per wing half—that are fixed to the fuselage square tube (Fig. 5c). The servomotor’s control input is transmitted through a ball joint linkage to the wing’s rear spar. Rotating the servo lever upward decreases the wing incidence angle, while rotating the servo lever downward increases the wing incidence angle (maximum incidence: ±10°, Fig. 2c).

The actuation of the tail folding and elevating are identical to our previous drone in^18^. We would like to refer you to this paper for a detailed description of these morphing mechanisms.

The weight ratios of the main components are listed in Fig. 5e.

### Wind tunnel setup

We studied the static aerodynamics of the folding and pitching wings in an open-jet wind tunnel (test volume: 1.92 × 1.68 × 1.5 m^3^, turbulence intensity: <1%, WindShape, Switzerland) at the expected mean flight velocity of 10 m/s (Reynolds number: 146,396). To measure the aerodynamic forces, we positioned the drone on an ATI Nano25 force and torque balance (National Instruments NI-DAQmx 9.5.1. logger, logging rate: 1000 Hz, averaging: 5) attached at the end of a cylindrical steel tube (l: 0.7 m, D: 0.025 m, d:0.02 m). The tube was attached to the handler of a Staubli TX-90 robotic arm with a custom made 3d-printed part and positioned in front of the wind tunnel. The robotic arm was programmed to systematically change the angle of attack of the drone (maximum angle error: <0.1°) with respect to the wind tunnel’s flow direction.

### Roll and yaw moment wind tunnel tests

We tested two different wing configurations (Fig. 3a): (i) left extended, right tucked, no wing pitching; (ii) left tucked and pitched upward, right tucked and pitched downward. The tail was set to tucked while the elevator and tail were set to their stick fixed position. We set the drone’s angles of attack between −4° and 20° in 4° steps.

For each angle of attack, we zeroed the the load cell at 0 m/s airspeed. Then, we set the wind tunnel airspeed to 10 m/s. Once the airspeed was reached, we started the recording of the *x* and the *z*-moment for 2 s (400 data points). From these measurements we calculated the mean roll moment*l*and yaw moment *n*, and their standard deviations. To respectively calculate the roll coefficient *C*_*l*_ and yaw coefficient *C*_*n*_, we used the equations from^2^ so that

1 $${C}_{l}=\frac{2l}{\rho bS{V}^{2}}$$

2 $${C}_{n}=\frac{2n}{\rho bS{V}^{2}},$$

where *S* = 0.224 m^2^ is the nominal wing area for all configurations, *b* = 1.18 m the wing span, *ρ* = 1.118 Kg/m^3^ the air density, and *V* = 10 m/s the airspeed^2^. The front propeller was removed during the wind tunnel measurements and thus our recording do not account for slipstream effects from the propulsion system. For consistency reasons, we used a cubic interpolation between the data points in Fig. 3b of the main text and provide the data points and their standard deviations in Supplementary Fig. S3.

### Roll performance flight experiment

To test the roll performance of wing pitching and wing folding during cruise flight, we performed two sets of tele-operated flight experiments. Each experiment was separated into two phases: the trim phase and the roll phase. During the trim phase, the pilot engaged a switch on the remote control, which set the throttle to 60 % and all the control surfaces to their stick-fixed position to fly a straight trajectory and obtain similar attitude and airspeed (~12 m/s) at experiment onset. Then, by engaging a second switch on the remote control, (i) the tucked wings are pitched (left: 10°, right: −10°), or (ii) the left wing is extended (45° to 130°), while the right wing remained tucked (45°) (Fig. 3a). Each experiment was performed four times (*n* = 4). To calculate the mean trajectories and their standard deviation of the angular rates (roll and yaw), as well as the angles (roll and yaw), we considered a 1 s time period after the switch was engaged. We used a linear interpolation to connect the position data points (250 Hz logging rate).

### Lift and pitch moment wind tunnel tests

We tested three different wing and tail configurations: (i) wings and tail tucked and the elevator deflected upward by −15° (Fig. 4a), (ii) wings tucked, tail extended and the elevator deflected upward by −15° (Fig. 4b), and (iii) wings and tail extended and the elevator deflected upward by −15° (Fig. 4a). All other control surfaces were in their stick-fixed positions. We tested an angle of attack range of −10° to 38° in 4° steps.

As for the roll and yaw moment wind tunnel experiments, we zeroed the the load cell at 0 m/s airspeed for each angle of attack. Then, we set the wind tunnel airspeed to 10 m/s. Once the airspeed was uniformly reached, we started the recording of the x-force *X*, z-force *Z*, and pitch moment *m* for 2 s (400 data points). To calculate the lift force for the *x*-force and *z*-force components, we used

3 $$L={{{{{\rm{sin}}}}}}(\alpha )X-{{{{{\rm{cos}}}}}}(\alpha )Z$$

with *α* being the angle of attack. To respectively calculate the lift coefficient *C*_*L*_ and pitch coefficients *C*_*m*_ from the recordings, we used the equations from^2^ so that

4 $${C}_{L}=\frac{2L}{\rho S{V}^{2}}$$

5 $${C}_{m}=\frac{2m}{\rho cS{V}^{2}},$$

where *L* was the lift force, *m* was the pitch moment, *S* = 0.224 m^2^ the nominal wing area for all configurations, $\bar{c}=0.22$ m the nominal mean aerodynamic chord, *ρ* = 1.118 Kg/m^3^ the air density, and *V* = 10 m/s the airspeed^2^. We did not account for slip stream effects again. For consistency reasons, we used a cubic interpolation between the data points in Fig. 4a to c of the main text and provide the data points and their standard deviations in Supplementary Fig. S4.

### Steady turn model

To better understand how the morphing capabilities of the morphing drone can improve the turn performance during a steady turn, we derived the equation of a steady coordinated turn lining the turn radius *R* with the lift coefficient *C*_*L*_. The three equation governing a steady turn (see^2^) are in the flight direction

6 $$T-D-W{{{{{\rm{sin}}}}}}(\gamma )=0,$$

in the radial direction

7 $$T{{{{{\rm{sin}}}}}}(\beta )+L{{{{{\rm{sin}}}}}}(\phi )-\left(\frac{W{V}^{2}{{{{{\rm{cos}}}}}}^{2}(\gamma )}{gR}\right)=0,$$

and in the direction normal to the radial and the flight direction

8 $$Lcos(\phi )-Wcos(\gamma )=0,$$

with *T* being the thrust force in N, *D* drag force in N, *L* the lift force in N, *W* the weight force in N, *β* the side slip angle in rad, *γ* the heading angle in rad, *ϕ* the bank angle in rad, *g* the gravitational acceleration in m/s^2^, *R* the radius in m, and *V* the airspeed in m/s. Assuming a steady level turn we can assume that the sideslip is zero (*β* = 0), there is no altitude change (*γ* = 0), which simplifies Eq. (6), (7), and (8) to

9 $$T-D=0$$

10 $$L{{{{{\rm{cos}}}}}}(\phi )-W=0$$

11 $$L{{{{{\rm{sin}}}}}}(\phi )-\frac{W{V}^{2}}{gR}=0.$$

Combining Eq. (10) and (11), we isolate the turn radius as

12 $$R=\frac{W{V}^{2}}{gL{{{{{\rm{sin}}}}}}(\phi )}=\frac{L{{{{{\rm{cos}}}}}}(\phi ){V}^{2}}{L{{{{{\rm{sin}}}}}}(\phi )g}=\frac{{V}^{2}}{g{{{{{\rm{tan}}}}}}(\phi )}.$$

The load factor is given as

13 $$n=L/W$$

and we know from Eq. (10) that *n* = *L*/*W* = 1/cos(*ϕ*). Given that the bank angle is constant we can replace $\tan (\phi )$ with $\sqrt{{n}^{2}-1}$, which leads to

14 $$R=\frac{{V}^{2}}{g\sqrt{{n}^{2}-1}}.$$

To make the Eq. (14) independent of the flight velocity, we can consider Eq. 4 and Eq. (13) to obtain

15 $$V=\sqrt{\frac{2nW}{\rho S{C}_{L}}},$$

which leads to the equation connecting the turn radius and lift coefficient such that

16 $$R=\frac{2W}{\rho gS{C}_{L,{{{{{\rm{turn}}}}}}}}\frac{n}{\sqrt{{n}^{2}-1}}.$$

### Banking turn flight tests

We performed a set of outdoor flights to demonstrate the role of avian-inspired wing and tail morphing during a turn maneuver (Supplementary Vid. S1). Each experiment was separated into three sub phases: trim, roll, and turn. To initialize the experiment, the safety pilot engaged a switch transmitter, which set the throttle to 60%, and all control surface in their stick-fixed position (wings and tail tucked). During the trim phase, the drone flew a straight line for about 2 s to maintain a similar initial attitude and airspeed (~12 m/s) conditions at turn maneuver onset between different trial runs. During the bank phase, we pitched the wing (left deflection: 10°, right deflection: −10°) to initiate a positive roll motion. All other control surfaces were set to their stick-fixed position. Then, once the required bank angle was reached we switched to the bank phase. In this phase, the wing pitching was set to zero again and either (i) the tucked tail was deflected upward by −15°, (ii) the tail was extended and deflected upward −15°, or both wing sides were extended, the tail was extended, and the tail was deflected upward −15° (Fig. 4a to c). Each configuration was flown ten times. The flights were performed in calm wind conditions on a large, open field.

To analyze the data, we first extracted the turn phases from the flight recordings. The banking turn phase was the period during which the LisEagle is in one of the aforementioned configurations (Fig. 4a to c). Thus, we considered the maneuver recordings when the drone reached the desired control output (which we selected manually a priori) until they left the desired control output again from the control output recordings. Once the relevant data was extracted, we rotated the position recordings of all the trial runs to have the same heading and same origin. Since we wanted to compare our flight test data with our steady-level turn model, we only considered trials with a small vertical displacement (<3 m). The largest common denominator of acceptable trial runs was five. Consequently, out of the ten flown trial runs, we selected the first five acceptable trial runs to calculate the mean turn radii and standard deviations. Reasons for the vertical displacements (and thus the deviation from a steady turn) were atmospheric turbulence and slight errors in bank angle given by the tele-operated nature of the experiment, and the reduction of the airspeed due to the constant throttle setting. To provide a continuous turn trajectory, we linearly interpolated between the position data points (10 Hz).

### Supplementary information

Supplementary Information

Description of Additional Supplementary Files

Supplementary Movie 1

## Data Availability

All data needed to evaluate the conclusions of the paper are available in the main manuscript and the Supplementary information.
