# Supplementary material for: Sharp turning maneuvers with avian-inspired wing and tail morphing
Source: Commun Eng. 2022 Nov 24;1:34. doi: 10.1038/s44172-022-00035-2 (PMC10956009; doi:10.1038/s44172-022-00035-2)
Supplement: Supplementary file 1 — Supplementary Information [file 44172_2022_35_MOESM1_ESM.pdf]

Supplementary Material for

Sharp turning maneuvers with avian-inspired wing and tail morphing

Enrico Ajanic, Mir Feroskhan, Valentin Wüest, and Dario Floreano

THIS PDF FILE INCLUDES:

- Supplementary Note 1. Asymmetric folding and pitching produce similar adverse yaw during cruise.
- Supplementary Note 2. LisEagle turn performance compared to other drones.
- Supplementary Fig. 1. Deflection response with respect to time for wing folding and pitching.
- Supplementary Fig. 2. Adverse yaw effects from asymmetric wing folding and pitching.
- Supplementary Fig. 3. Data points and standard deviation (n = 400) of the roll coefficient and the yaw coefficient.
- Supplementary Fig. 4. Data points and standard deviation (n = 400) of the lift and pitch coefficients.
- Supplementary Fig. 5. Mean load factor, bank angle, and airspeed during the banking turn flight for the three different configurations.
- Supplementary Fig. 6. Comparison of modeled turn radius of the LisEagle and a commercial drone called Bixler.
- Legend to the Supplementary video.
- Supplementary References.

SUPPLEMENTARY NOTE S1. ASYMMETRIC FOLDING AND PITCHING PRODUCE SIMILAR ADVERSE YAW DURING CRUISE

Adverse yaw is caused by a drag asymmetry between the two wing sides (Supplementary Fig. 2A, left). For example, assuming a drone flies in steady state with an angle of attack of 4°. If we want to roll to the right and pitch the left wing upward by 4°, and the right wing downward by 4°, then the wings' local angle of attack on the left wing is 8°, and on the right wing 0°. Increasing the angle of attack increases lift and drag, while decreasing the angle of attack decreases lift and drag. Consequently, the aerodynamic forces on the left wing are greater than on the right wing, causing a clockwise rolling motion, but also a counter clockwise yawing motion [1].

This yawing motion causes a sideslip angle during the bank phase also called slipping (Supplementary Fig. 2A, right), which causes an unwanted a sideway force. This force can be split into a drag, centrifugal, and lift component. The increased centrifugal force leads to an increase in turn radius, while the increased drag force reduced the aerodynamic efficiency. Thus, to keep the slipping small, the adverse yaw must be minimized during the roll phase.

Here, we want to provide the adverse yaw measurements (Supplementary Fig. 2C to E) that were collected during the roll phase study in the main manuscript for asymmetric folding and pitching (Fig. 3). Our adverse yaw wind tunnel measurements indicate that the adverse yaw moment is of similar magnitude when folding or pitching asymmetrically during cruise flight (Supplementary Fig. 2C and D, red line) although the roll moment is much larger for pitching (Fig. 3C). We also detect an increase in adverse yaw when pitching with increasing angles of attack, while when folding the adverse yaw decreases with increasing angles of attack. When folding or pitching during flight, we see that the adverse yaw moment leads to an initial adverse yaw motion for both tested configurations (Supplementary Fig. 2D) and a slight slipping (negative yaw angle) (Supplementary Fig. 2E).

SUPPLEMENTARY NOTE S2. LISEAGLE TURN PERFORMANCE COMPARED TO OTHER DRONES

Using the turn model (Eq. 16), it is possible to compare different drones, given the wing area, mass and maximum lift coefficient. To compare the LisEagle's turn performance with another drone, we consider a commercial drone called Bixler with a similar wing span (1500 mm) and mass (1 kg, with autopilot avionics) as the LisEagle (1440 m, 0.711 kg). From literature, we can also obtain the Bixler's maximum lift coefficient ($C_{L,max} = 1.25$ [2]). Given a similar wing loading (LisEagle: 31.2 N/m^3, Bixler: 32 N/m^3), the curves are nearly the same, yet while the Bixler achieves a maximum lift coefficient of 1.25, we measured a maximum lift coefficient of 1.68, which leads to a minimum (ideal) turn radius of 4.9 m and 3.6 m, respectively (Supplementary Fig. 5). It is important to note that the LisEagle's lift coefficient obtained with the extended wing and tail configuration (Fig. 4C) does not represent the maximum lift coefficient. Deflecting the tail further upward might further increase the lift coefficient, which further reduces the

minimum turn radius. Furthermore, it is important to note that such comparisons oversimplify the complex performance characteristics of our morphing drone. As discussed in our previous study [3], wing and tail morphing also allows to notably reduce drag during cruise flight and change the pitch stability, which the Bixler cannot do.

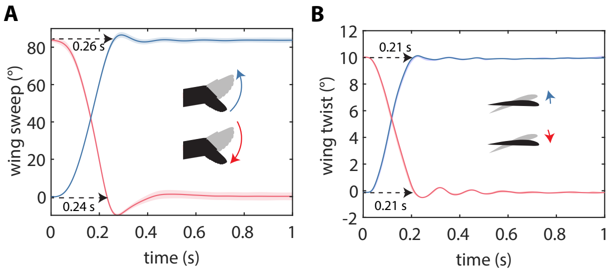

Supplementary Fig. S 1. Deflection response with respect to time for wing folding and pitching. To obtain LisEagle's wing folding and pitching change with respect to time, we tracked the wing motion using our motion capture system (logging rate: 240 Hz) consisting of 25 cameras and taped three 16 mm ball markers on the main wing and the wrist. We performed two sets of experiments four times each (n =4): (A) we extended the wing from 45° to 130° and from 130° to 45°, which lead to a rise time of 0.26s and an overshoot of 5° when extending and a fall time of 0.24 and an undershoot of 9° when tucking. (B) We also tucked the wing and twisted the wing from its neutral position at 0° to 10° and from 10° to 0° pitch, which lead to a rise time of 0.21 s and an overshoot of 0.1° and a fall time of 0.21 and an undershoot of 0.2°.

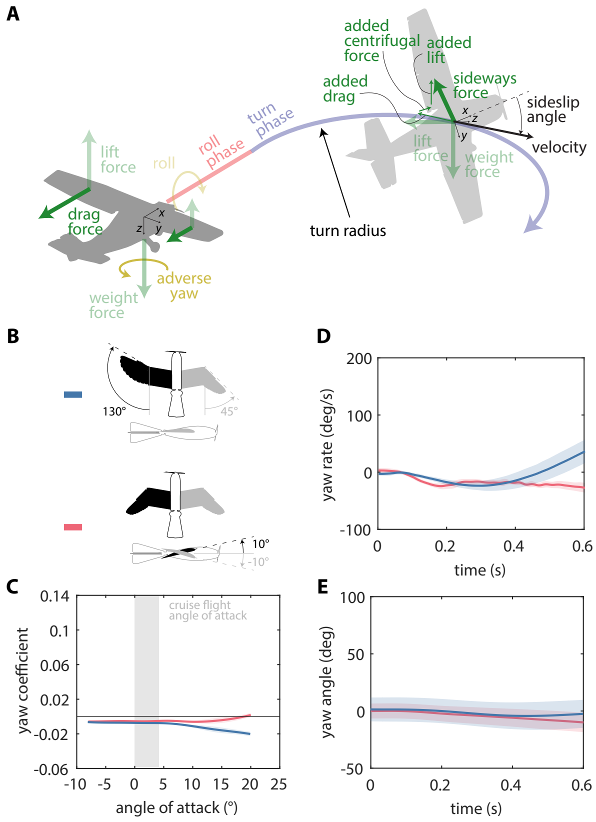

Supplementary Fig. S 2. Adverse yaw when turning. The shaded regions depict the standard deviation of four trial runs (n = 4). (A) Illustration of the formation of the adverse yaw moment and the thereto linked slipping when banking. (B) We tested two configurations: Asymmetric wing folding and asymmetric wing pitching. (C) Adverse yaw moment measurements in the wind tunnel for the two tested configurations. (D) Yaw rate during outdoor flight tests for the two tested configurations. (E) Yaw angle when flying for the two tested configurations.

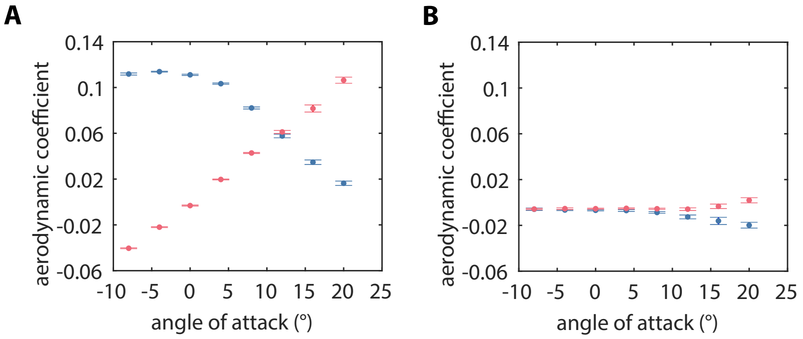

Supplementary Fig. S 3. Mean and standard deviation (n = 400) of the roll coefficient and the yaw coefficient wind tunnel measurements. (A) Measured data points in the wind tunnel and standard deviation of the roll coefficient when applying wing folding (blue) and pitching (red). (B) Measured data points in the wind tunnel and standard deviation of the yaw coefficient when applying wing folding (blue) and pitching (red).

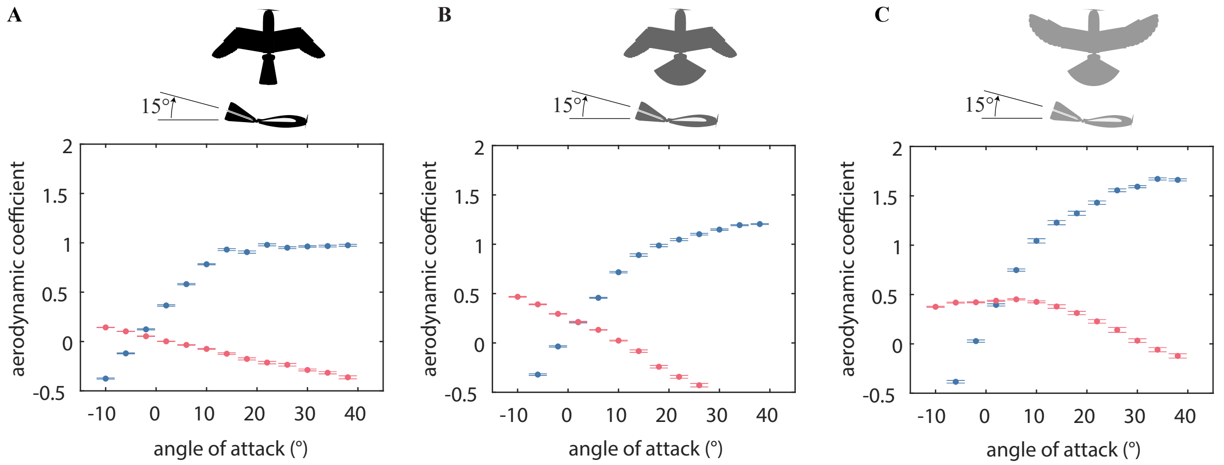

Supplementary Fig. S 4. Mean and standard deviation (n = 400) of the lift and pitch coefficients wind tunnel measurements. (A) Measured data points in the wind tunnel and standard deviation of the lift (blue) and pitch (red) coefficients when applying tucked wing and tail, and an elevator deflection of -15°. (B) Measured data points in the wind tunnel and standard deviation of the lift (blue) and pitch (red) coefficients when applying tucked wing and extended tail, and an elevator deflection of -15°. (C) Measured data points in the wind tunnel and standard deviation of the lift (blue) and pitch (red) coefficients when applying extended wing and tail, and an elevator deflection of -15°.

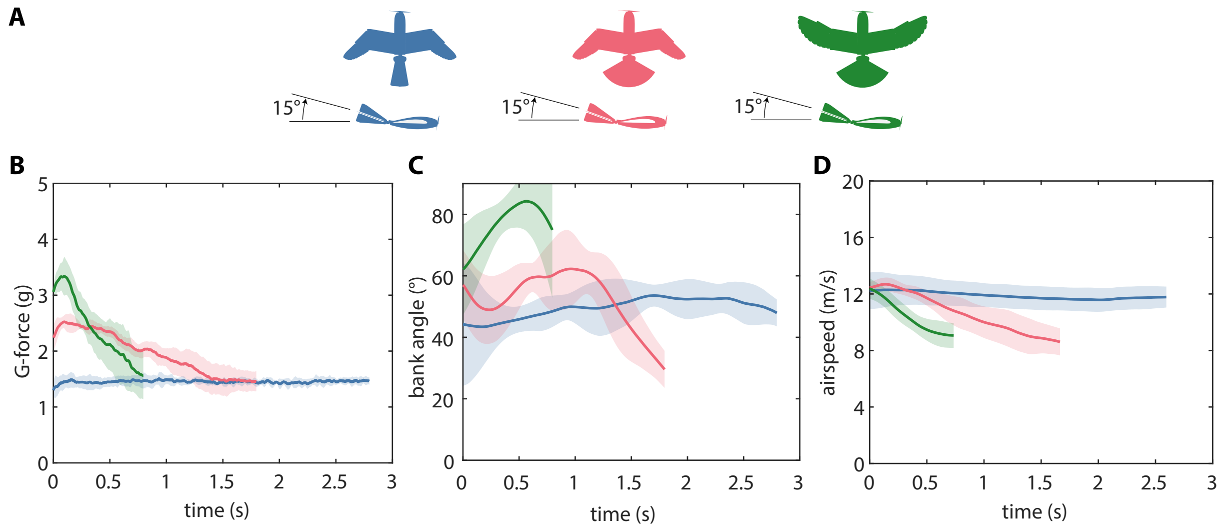

Supplementary Fig. S 5. Mean g-force, bank angle, and airspeed during the outdoor banking turn flight tests for the three different configurations. The shaded region indicates the standard deviation of five different trial runs (n=5). (A) We tested tucked wing, tucked tail (blue); tucked wing, extended tail (red); extended wing, extended tail (green). (B) The instantaneous g-force calculates as the two-norm of all body accelerations divided by the gravitational acceleration $g = 9.81\text{m/s}^2$, and (C) the bank angle are highest for the extended wing and tail configuration. (D) Due to the constant thrust during the maneuver, we see a reduction in flight speed for the extended wing and tail configuration and the tucked wing and extended tail configurations, presumably due to the increased drag from the higher angle of attack flight and the incrased lifting surfaces.

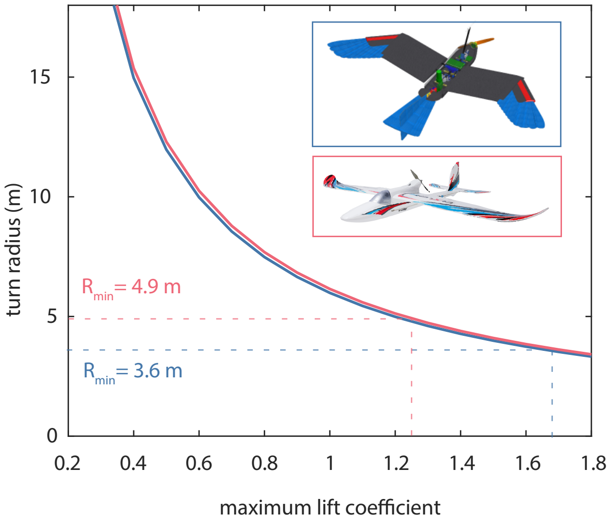

Supplementary Fig. S 6. Comparison of modeled turn radius (Eq. 16) of the LisEagle and a commercial drone called Bixler. Given the similar wing loading (LisEagle: 31.2 N/m^3, Bixler: 32 N/m^3) and a load factor of $n_{lf} = 3$, the LisHawk drone (blue inset and line) can achieve a minimum radius of 3.6 m (considering the configuration of Fig. 4C), while the Bixler (red inset and line) achieves a minimum turn radius of 4.9 m. The maximum lift coefficient was taken from [2].

SUPPLEMENTARY VIDEO S1. IN-FLIGHT BANKING MANEUVER.

Banking maneuver for all three cases of tucked wing and tail, tucked wing and extended tail, and extended wing and tail to show the reduction in turn radius due to morphing.

SUPPLEMENTARY REFERENCES

[1] Robert C. Nelson. *Flight stability and automatic control*. McGraw Hill, Boston, 2 edition, 1998.

[2] Colin Greatwood, Antony Waldock, and Thomas Richardson. Perched landing manoeuvres with a variable sweep wing UAV. *Aerospace Science and Technology*, 71:510–520, 2017.

[3] Enrico Ajanic, Mir Feroskhan, Stefano Mintchev, Flavio Noca, and Dario Floreano. Bioinspired wing and tail morphing extends drone flight capabilities. *Science Robotics*, 5(47), 2020.
