# Supplementary material for: Sharp turning maneuvers with avian-inspired wing and tail morphing
Source: Commun Eng. 2022 Nov 24;1:34. doi: 10.1038/s44172-022-00035-2 (PMC10956009; doi:10.1038/s44172-022-00035-2)
Supplement: Supplementary file 2 — Description of Additional Supplementary Files [file 44172_2022_35_MOESM2_ESM.docx]

Description of Additional Supplementary Files

**File name:** Supplementary Video 1

**Description:** Video describing and visualizing the banking turn experiment.
